# Post-myocardial infarction treatment with resiniferatoxin modulates the expression of important genes involved in inflammation, plaque stability and angiogenesis

**DOI:** 10.15190/d.2023.2

**Published:** 2023-03-31

**Authors:** Andrei Alexandru Mircea

**Affiliations:** ^1^Carol Davila University of Medicine and Pharmacy, Bucharest, Romania; ^2^UCLA Health Arrhythmia Center, Los Angeles, CA 90095, USA

**Keywords:** Myocardial infarction, TRPV1, RNA sequence analysis, resiniferatoxin, LIPG, pentraxin 3, ADAM thrombospondinin family.

## Abstract

Ventricular tachycardia (VT) and ventricular fibrillation (VF) are the most frequent causes of death in the first 24 hours after myocardial infarction. Previous studies showed that depleting TRPV1 receptors with resiniferatoxin (RTX) led to a reduced risk of VT and VF post-myocardial infarction. Therefore, the question of resiniferatoxin as a cardioprotector against myocardial infarction (MI)-induced VT and VF was raised. The RNA sequence data from 3 groups of pigs, each having 4 animals (4 controls, 4 myocardial infarction - MI, and 4 RTX + MI) was analyzed through the lens of differentially expressed genes. The differential expression comparison was conducted in two ways: MI versus Control and RTX+MI versus MI. The results showed the downregulation of deleterious genes involved in inflammation and future plaque instability in the RTX group compared with the MI group. In the case of some of the genes, these findings were reinforced by obtaining the same trends in the MI versus Control group. All in all, we propose further investigation of RTX as a prophylactic method against cardiovascular complications of MI.

## INTRODUCTION

Cardiac transient receptor potential channel 1 (TRPV1) afferent signaling plays a key role in arrhythmogenesis post-myocardial infarction. TRPV1 is a receptor located on the afferent fibers and it partly mediates the cardiac sympathetic afferent reflex (CSAR)^1^. TRPV1 is activated by heat, capsaicin, metabolites of ischemia (e.g., bradykinin), and mechanical transduction^2^. During and after a myocardial infarction the CSAR is activated, contributing to the persistence of reflex adrenergic activation. This enhances ventricular tachycardia (VT) or ventricular fibrillation (VF) occurrence.

Interestingly, other data on capsaicin-induced activation of TRPV1 in cardiomyocytes enhanced their apoptosis, presumably via increased intracellular levels of calcium following TRPV1 stimulation^3^. Another way to induce apoptosis was represented by the higher than normal mitochondrial superoxide levels^3^. In contrast, adding a TRPV1 antagonist such as capsazepine led to improved cell viability following hypoxia and ischemia of the heart^3^.

It is proposed that altering the cardiac sympathetic afferent reflex post-myocardial infarction (MI) will improve the prognosis of patients. After MI, there is augmented CSAR^4^. A study conducted in 2019 revealed that the denervation of the afferent component reduced the number of ventricular tachycardia (VT)/ventricular fibrillation (VF) experienced^4^. This finding was related to the suppression of stellate ganglion activity, and presumably, cardiac adrenergic tone. In turn, the electrophysiologic stability of the myocardium was enhanced, protecting the heart from developing VT/VF.

Resiniferatoxin (RTX) depletes TRPV1 in the epicardium through direct application, exerting beneficial effects post-MI. A previous experiment found that CaMK II and RYR2 channel expression was reduced in subjects with HF treated with RTX^5^. Both are associated with impaired ventricular function. Furthermore, subjects with MI present with higher than normal tyrosine hydroxylase, connexin 43, TGF β1, and growth-associated protein 43^6^. Such markers are associated with cardiac remodeling and electrical instability of the heart. In the case of subjects treated with RTX after the MI episode, these four variables were lower than expected. Consequently, improvements in the LV function and the ventricular electrophysiologic properties were recorded^6^.

How cardiac afferent depletion by RTX impacts myocardial gene expression to impart its beneficial effects remains unknown.

## MATERIALS AND METHODS

The study was performed with the required approval from the host institution, using three groups of pigs, each group containing 4 subjects. The first group is a control group. The second one had a MI event induced. Lastly, the third group got both a MI and an RTX treatment. The cardiac tissue was obtained after cardiac dissection. The 12 samples were randomly arranged (control, infarcted, and treated-infarct animals). The specific pathways of interest researched were the GPCR signaling pathways for both M2 signaling pathway as well as the beta 1 pathway, the ion channel expression and regulation, the myocardial stress response pathways, and the inflammation pathways. The purification method used was a total RNA isolation using Qiagen RNAeasy Column. The concentration was measured via Nanodrop.

The data was obtained through Illumina sequencing (HiSeq 3000), which uses sequencing by synthesis mechanism. The kit used for the RNASeq libraries was a KAPA Stranded RNA-Seq Kit. A flow cell had the adapter (sequence primer) bound to the original sequence. After the polymerase elongated the complementary chain (i.e.: the reverse strand), the original strand was washed away. Now, the sequence primer complementary to the other end of the reverse strand caused the bending over and bridge amplification, leading to a forward strand alongside the reverse strand. This process was repeated until the PCR colony was obtained. Lastly, a sequence primer was added to the cluster. The cluster was stimulated by a light source and a fluorescent color was emitted each time the 4 competing nucleotides were added to the complementary strand. The depth of coverage was two-lane for all, with approximately 50 million reads. The read length was 1x50. The data quality check was done with Illumina SAV.

**Figure 1:**
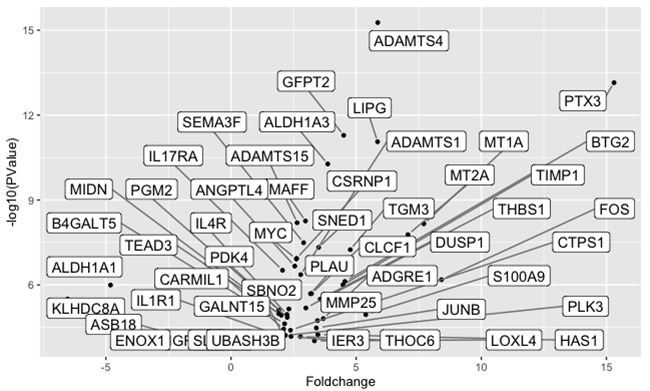
RTX+MI vs. MI - differentially expressed genes

**Figure 2:**
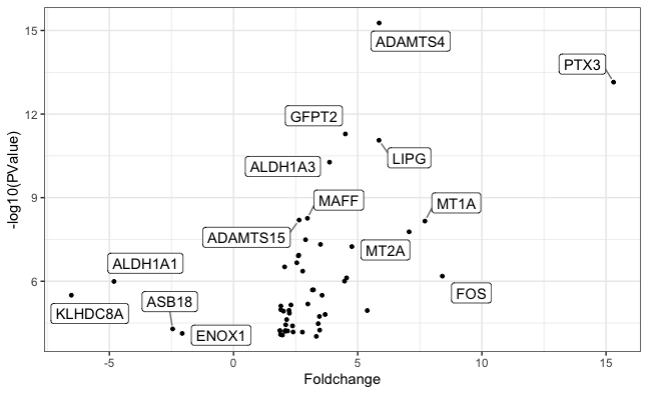
Outliers in the differential gene expression analysis of RTX+MI vs. MI

**Figure 3:**
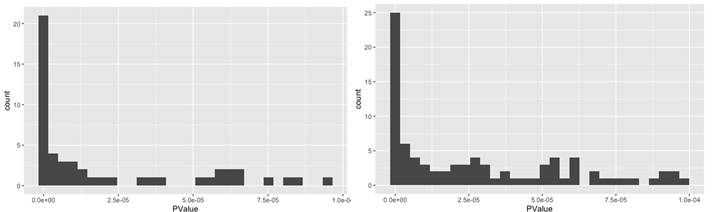
Gene Counts for MI+RTX vs. MI and MI vs. Control (A) MI + RTX vs. MI Counts of genes with P-values below 10e-5 (B) MI vs. Control Counts of genes with P values below 10e-5.

The data processing consisted of two major steps. Firstly, the data mapping was carried out using Bowtie2 version 2.1.0. Secondly, the gene expression level was estimated with RSEM v1.2.15. The gene expression was normalized using a trimmed mean of M-values. The results identified differentially expressed genes using EdgeR. The genes were filtered for a a fold change of > 1.5 and a p-value > 1 * 10e-5. RStudio was employed for the differentially expressed genes (Figure 1), for data visualization of outliers (Figure 2), and for the generation of heatmaps (Figure 3). The pathway enrichment analysis was conducted with the support of the gProfiler database. To better grasp the outliers and the genes differentially expressed in the RTX+MI vs. MI comparison, RStudio was used for data visualization. The ggplot library was part of the code (Figure 1 and Figure 2).

There were 85 differentially expressed genes (Figure 1) in the comparison between the MI and the control group, with a P value greater than 10e-5. In contrast, in the comparison conducted between the MI group and the MI group treated with resiniferatoxin only 51 genes had a P value greater than 10e-5 which were differentially expressed (see Supplementary Information). Some genes or families of genes were found differentially expressed in both comparisons, requiring further investigation. Table 1 below summarizes them. Moreover, tumoral genes which have an effect on cardiovascular disease were found to change in the RTX+MI group in comparison with the MI group (Table 2). The outliers in the DGE of RTX+MI vs. MI were plotted (Figure 2), alongside the pathways they are involved.

**Table 1:** Family of genes and genes differentially expressed in-between groups and common to both comparisons

Gene	RTX+MI vs. MI		MI vs. Control	
	Fold change	P value	Fold change	P value
LIPG	5.85	8.72*10e-12	-2.58	1.56e-06
ANGPTL4 vs. ANGPT2	2.54	2.16e-07	-1.87	9.49e-06
ADAMTS (-1&-4 vs. -2)	2.78 & 5.86	4.34e-07 & 5.37e-16	2.63	2.81e-07
MMP (-25 vs. -15)	3.45	1.82e-05	2.23	1.96e-05

**Table 2:** Tumoral genes differentially expressed in RTX+MI vs. MI comparison

Tumoral genes	Foldchange	P-Value
CSRNP1	2.62	1.158e-07
MYC	2.61	1.219e-07
FOS	8.40	6.554e-07

## RESULTS

There were 85 differentially expressed genes (Figure 1) in the comparison between the MI and the control group, with a P value greater than 10e-5. In contrast, in the comparison conducted between the MI group and the MI group treated with resiniferatoxin only 51 genes had a P value greater than 10e-5 which were differentially expressed (see Supplementary Information). Some genes or families of genes were found differentially expressed in both comparisons, requiring further investigation. Table 1 below summarizes them. Moreover, tumoral genes which have an effect on cardiovascular disease were found to change in the RTX+MI group in comparison with the MI group (Table 2). The outliers in the DGE of RTX+MI vs. MI were plotted (Figure 2), alongside the pathways they are involved.

## DISCUSSION

Our study opens new questions. Resiniferatoxin seemed to modulate expression of both deleterious and beneficial genes involved in the complications following myocardial infarction. Thus, we discussed which genes of interest need further investigation to prove the beneficial effects of resiniferatoxin post-MI.

### Family of genes and genes commonly found in both differential expression analyses

Between the genes differentially expressed in both comparisons (MI+RTX vs. MI and MI vs. control), we found lipase G, endothelial type (LIPG) is an enzyme that plays a key role in both atherosclerosis and oncogenesis. Its deleterious effects on atherosclerosis have been proven previously^7^. Moreover, patients with atherosclerosis have high levels of LIPG expression^7^. Its role in inflammation is described by LIPG's relationship with cytokines. More precisely, IL6 is a potent activator of atherosclerosis, while being an up regulator of LIPG^8^. On the other hand, previous studies used LIPG inhibitors to prevent and treat cardiovascular diseases^9^. The therapeutic result was an increased expression of cardioprotective HDL-C^9^.

Our research results reveal that RTX modulates LIPG expression, which in turn may potentially explain the previously observed beneficial roles of RTX, through a cardioprotective role. In our MI group, an almost 6-fold increase in LIPG expression was measured in comparison with the RTX+MI group. On the other hand, the control group had 2.58-fold lower levels in their LIPG expression, demonstrating the role of MI in upregulating LIPG expression.

ADAMTS-1, alongside ADAMTS-4,-5,-8, and -15, is one of the proteoglycanases responsible for cleaving proteoglycans^10^. ADAMTS-1 is responsible for cleaving versican, a proteoglycan of early and late atherosclerotic disease. Its cleavage allows vascular smooth muscle cells to invade the intima and proliferate, leading to neovascularization. Moreover, its decomposition compromises the fibrous cap integrity^11-13^. Overexpression of ADAMTS-1 was found with the neointimal formation in a murine model of carotid artery stenosis^14^. In a previous GWAS, the heterozygous and homozygous genes encoding for a proline amino acid were shown to increase the risk of an acute coronary syndrome^15^. It was shown that pravastatin therapy reduced considerably the risk in these patients.

RTX modulates ADAMTS-1 in patients that suffered from myocardial infarction. Our findings revealed that RTX-treated subjects post-MI had a 2.78-fold decrease in ADAMTS1 in comparison with the MI subjects. This suggests a potentially protective role of RTX similar to pravastatin for future ACS, although further experiments need to be performed for validation.

ADAMTS-2 plays a key role in cardiac remodeling, functioning as a procollagen N-propeptidase^16^. It was found that patients with HCM have ADAMTS-2 upregulated to promote myocardial repair and scarring^17^. However, overexpression of ADAMTS-2 leads to inhibition of the PI3K/AKT pathway involved in cardiac remodeling. In our cohort, the MI patients had increased gene expression levels of ADAMTS-2 in comparison with the controls, indicating remodeling impairments.

Our findings suggest an almost 6-fold increase in ADAMTS-4 in patients with MI without the RTX treatment. ADAMTS-4 is a proteinase responsible for the degradation of versican, an extracellular matrix protein. Recent reports indicate a higher ADAMTS-4 expression in patients with unstable atherosclerotic plaques as well as ACS^18-22^*.* A previous baboon aortoiliac graft study identified the role ADAMTS-4 plays in vascular smooth muscle cell death^23^. Therefore, the decreased ADAMTS-4 expression of the RTX-treated pigs correlates with a reduced vascular smooth muscle cell death. In a mice model, ADAMTS-4 knock-out mice showed less lipid and macrophage accumulation with a more stable plaque, comprising a thicker fibrous cap with more collagen deposition^24^. Since RTX has a similar effect to ADAMTS-4 reduction, RTX could be translated into the clinic as a plaque stabilizer for preventing postMI complications.

Another pair differentially expressed in the two comparative groups was the angiopoietin family. In the RTX+MI vs. MI comparison, the ANGPTL4 gene was found to be 2.54 fold more expressed in the MI group. Inactivating mutations in ANGPTL4 led to lower levels of triglycerides and higher levels of HD^18^. Moreover, monoclonal antibody inhibition of ANGPTL4 in monkey and rat models significantly reduced triglycerides. All in all, inactivating variants of ANGPTL4 are associated with reduced CAD in humans^18^.

ANGPT2 is the other member of the angiopoietin family that showed a differential expression in the MI group in comparison with the control group. In the literature, ANGPT2 transcription is usually elevated in endothelial cells at the infarct border zone^19^. Its expression enhances hypoxia and neutrophil infiltration as well as vascular permeability. Our findings suggest an upregulation of the ANGPT2 gene in the MI group. The control group had a -1.84 fold change in its expression.

All in all, resiniferatoxin seems to modulate genes involved in CAD and the atherosclerotic plaque. Thus, it could play a role in modulating reinfarction following stent treatment.

### Possible resiniferatoxin effects on cardiovascular gene expression

Although it remains to be proved whether RTX had a significant clinical effect, the differential gene expression analysis between the MI and MI+RTX group highlighted significant differences in gene expression for genes linked to cardiovascular disease.

A pair of highly expressed transcripts in the MI group in comparison with the RTX+MI group was MMP 25 and TIMP1. Research shows that pharmacologic inhibition of MMPs contributes to LV dilation attenuation. This process was proposed as a potential therapy for HF after MI^20^. TIMP1 plays two key roles in the relationship with MMPs. Firstly, TIMPs inhibit MMPs^21^. Secondly, TIMP1 inhibits angiogenesis required for revascularization of the infarcted area^21^. Our research shows that patients with MI had a 3.45-fold increase in the expression of MMP 25 and a 4.46-fold higher level of TIMP1. These findings may potentially suggest that neovascularization is more impaired in the MI group, although further experiments are required to have a definitive conclusion.

A potent and beneficial effect resiniferatoxin exerts on PTX3 gene expression. Pentraxin 3 is responsible for angiogenesis and tissue remodeling. The PLATO trial^25^ showed that admission serum levels of PTX3 are correlated with cardiovascular death and spontaneous MI. PTX3 has a higher sensitivity than CRP and a strong association with NT-proBNP and troponin T levels. Thus, as resiniferatoxin subjects showed a 15-fold decrease, RTX could be used as a lowering agent during the admission process for a STEMI.

The HUNT2 study^26^ highlighted the role PTX3 had as a biomarker for a first MI event in a study population observed for 10 years. There are already trials that use other agents, such as rosuvastatin, to decrease PTX3^27^. The literature on PTX3 finds that estimating the cardiovascular mortality risk with PTX3 and NT-proBNP increases the accuracy of the estimation^28^. Thus, we propose a future study to verify the translation of the resiniferatoxin benefits into a protocol consisting of PTX3 determination and cardiovascular risk evaluation, followed by RTX treatment for patients at high risk of a first MI event.

Under hypoxic strain, cardiomyocytes undergo activation of the Wnt/β-catenin pathway. The ortholog in humans of CSRNP1 is Axud1^29^. Axud1 is a proapoptotic protein. In the literature, Axud1-knockdown mice had less activation of the Wnt/β-catenin pathway and reduced apoptosis^30^. Thus, by reducing the levels of CSRNP1, RTX may have some inhibitory roles on apoptosis following MI that needs to be further investigated.

A previous differential gene expression study demonstrated higher levels of FOS expression in patients who developed HF post-MI compared to patients who had a MI without HF^31^. Since in the RTX+MI group the expression was lower, these subjects can be protected against future HF. FOS was found to be upregulated in hypertrophied infarcted myocardium^32^. As such RTX decreasing FOS might provide a protective role against excessive hypertrophy following MI, an idea which needs further validation.

A previous study highlighted how miR-142-5p and miR-212-5p regulate the c-MYC pathway during myocardial infarction^33^. The overexpression of miRNA led to the suppression of proliferation and collagen formation. Thus, the mice which had a hypoxic-ischemic injury improved their heart function. Since RTX suppresses MYC as miR-142-5p and miR-212-5p do, it can be hypothesized that RTX improves cardiac function through suppression of c-Myc, a hypothesis that needs to be further studied.

Overexpression of THBS1 (thrombospondinin-1) was shown in literature to have deleterious cardiac effects and its expression was inhibited by RTX. A differential gene expression study^34^ identified THBS-1 as a candidate biomarker for cardiac hypertrophy. Moreover, THBS-1 gene was overexpressed in endothelin-stimulated cardiomyocytes, leading to hypertrophy. Another instance when THBS-1 was overexpressed was in the case of autophagy-mediated cardiac atrophy^35^. To corroborate these aspects with our data, RTX might protect against excessive hypertrophy post-MI as well as against cardiomyocyte atrophy following infarction. The MI group had a 3.56 fold increase in the THBS1 expression in comparison with the RTX+MI group. Further investigation is warranted to verify this hypothesis.

RTX has an effect on the extracellular space, by reducing the expression of SEMA3F (2.9 fold). The previous research^36^ showed that SEMA3F has a key role in upregulating PECAM1 and leukocyte extravasation. Furthermore, the serum levels of cardiac arrest patients were found to be elevated after cardiac arrest. These levels were associated with decreased survival, myocardial dysfunction and prolonged vasopressor activity. By modulating SEMA3F, RTX could potentially reduce the risk of HF, prolong survival, prevent fatal arrhythmias that cause cardiac arrest and most certainly reduce the inflammation after MI.

RTX impacts the NAD+ pathway as well. In vitro studies^37^ identified that highly expressing ALDH1A3 cardiomyocytes are prone to excessive proliferation into the atrium. These cells were collected from patients with ischemic disease. By decreasing 3.86 fold, RTX could potentially protect against the electrical instability of the atria post-MI, a hypothesis which remains to be further investigated.

## CONCLUSION

All in all, resiniferatoxin modulates the expression of important genes that are potentially related with beneficial outcomes following myocardial infarction. By downregulating the expression of genes involved in inflammation, plaque instability, and inappropriate angiogenesis, resiniferatoxin is a potential prophylactic method for future ACS in myocardial infarction patients. Additional detailed investigations have to be performed to confirm the beneficial role of the indicated gene expression changes post-MI.
